# Evolving Treatment Strategies for Systemic Lupus Erythematosus in Clinical Practice: A Narrative Review

**DOI:** 10.7759/cureus.75062

**Published:** 2024-12-03

**Authors:** Alan D Kaye, Joseph P Tassin, William c Upshaw, Chandni R Patel, Alison M Hawkins, Caroline R Burroughs, Kristin Nicole Bembenick, Chizoba N Mosieri, Shahab Ahmadzadeh, Adam M Kaye, Sahar Shekoohi, Giustino Varrassi

**Affiliations:** 1 Anesthesiology, Louisiana State University Health Sciences Center, Shreveport, USA; 2 School of Dentistry, Louisiana State University Health Sciences Center, New Orleans, USA; 3 School of Medicine, Louisiana State University Health Sciences Center, Shreveport, USA; 4 School of Medicine, St. George’s University, West Indies, GRD; 5 Pharmacy Practice, Thomas J. Long School of Pharmacy and Health Sciences University of the Pacific, Stockton, USA; 6 Pain Medicine, Fondazione Paolo Procacci, Rome, ITA

**Keywords:** autoimmune disease, monoclonal antibodies, new treatment, sle, systemic lupus erythematosus

## Abstract

Systemic lupus erythematosus (SLE) is an autoimmune disease that more commonly affects African American people, although it is seen in people of all racial backgrounds. This condition is characterized by a dysregulated immune response resulting in widespread inflammation. Clinical manifestations caused by this inflammation include arthritis, anemia, cutaneous rashes, pleuritis, and nephritis. Treatment for SLE aims to reduce disease activity and maintain a state of low inflammation. In this regard, numerous treatments are used, such as hydroxychloroquine, glucocorticoids, and non-glucocorticoid immunosuppressants such as methotrexate. Related to these drugs resulting in significant adverse effects and being ineffective in controlling SLE symptoms in some patients, new biologic agents have been created in hopes of better treating SLE. This includes belimumab and anifrolumab, monoclonal antibodies directed against the cytokine, and type 1 interferon receptor, respectively. These agents are indicated in patients with SLE whose symptoms are inadequately controlled by standard therapy alone. Clinical trials have shown that these agents effectively reduce SLE symptoms as judged using standardized metrics of disease activity. These biological agents have also been shown to have generally mild side effects when taken by patients with SLE, making them safe for use. In addition to the above medications, new treatments are being developed for SLE patients, such as cenerimod, litifilimab, chimeric antigen receptor T cells, and DS-7011a (anti-toll-like receptor 7 monoclonal antibody). These new treatments have shown promise in clinical trials. However, more information regarding their safety and efficacy is needed before they are available for the treatment of SLE.

## Introduction and background

Systemic lupus erythematosus (SLE) is an autoimmune condition that affects approximately 144 per 100,000 people. SLE is six times more common in women than in men and disproportionately affects African American people [1]. The disease leads to a high economic burden on those with the condition, costing over 50,000 dollars in medical costs annually for those with severe forms of SLE [2]. In terms of the etiology of SLE, evidence shows that an essential genetic component plays a role in its development, with monozygotic twin studies revealing a concordance rate as high as 24% [3]. Specific genes linked with the development of SLE include certain alleles of the human leukocyte antigen-DR2 (HLA-DR2) and HLA-DR3 genes, the Fc gamma receptor III gene, and the gene encoding for the C1q protein [4-6]. Other factors believed to play a role in the development of lupus include viral infections, certain drugs, exposure to high levels of estrogen, and smoking [7].

The pathophysiology of SLE is complex and involves numerous components. Critical to developing SLE is the formation of autoantibodies such as antinuclear antibodies, anti-double stranded DNA antibodies, and anti-Sm antibodies. These immune complexes may deposit in tissues and cause the activation of the complement cascade, resulting in inflammation of the affected organs [8]. The importance of this phenomenon in the pathogenesis of SLE can be seen in how levels of specific autoantibodies correlate with levels of disease activity [9]. An abnormal adaptive immune system also characterizes SLE. Specifically, patients with SLE have been shown to have more B lymphocytes than healthy patients with a heightened sensitivity to IL-6, which promotes the production of IgG antibodies [10]. Additionally, patients with SLE tend to have more IL-10 expression than those without the condition, which promotes B-cell proliferation and activation [11]. SLE patients also have an abnormally low expression of IL-12, which results in an enhanced humoral immune response [12].

Initially, patients with SLE present with constitutional symptoms, e.g., fatigue, malaise, fever, and weight loss. Later complications and symptoms include inflammatory polyarthritis, anemia of chronic disease, pleuritis, pericarditis with associated effusion, esophageal dysmotility, and recurrent spontaneous abortions due to patients being in a hypercoagulable state [7]. Lupus nephritis is a particularly severe manifestation of this condition, affecting approximately half of SLE patients [13]. It involves the deposition of immune complexes in the kidney; different subtypes of lupus nephritis are classified based on the location of the deposited immune complexes in the glomerular filtration barrier. Ultimately, lupus nephritis may lead to significant proteinuria, hypertension, and edema due to hypoalbuminemia and may progress to kidney failure if not adequately treated [14].

Treatment for SLE is primarily based on achieving a state of low disease activity [15]. The pharmacological agents used to achieve this goal include glucocorticoids (GCs), non-GC immunosuppressants, hydroxychloroquine (HCQ), and newer biologic agents [16]. This article describes the drugs used to treat lupus. Emphasis is placed on the mechanism of action of these drugs, adverse effects, and indications for their use in patients with SLE.

## Review

Nonbiologic treatments for SLE

The treatment and management of SLE have three main goals: preventing or suppressing flares, reducing organ damage, and minimizing the adverse effects caused by immunosuppression. Nonbiologic drugs such as antimalarials, GCs, and various non-corticosteroid immunosuppressants (NCIs) often achieve these goals. A combination of these drugs has long been the standard of care in the treatment of SLE [17-19].

Antimalarials are often used in the treatment of SLE [16]. Initially developed for the treatment of plasmodium infections, antimalarials, more specifically HCQ, have since become one of the most valuable first-line treatments for SLE. Currently, HCQ is recommended for use in all SLE patients, as there are no contraindications [19]. HCQ can reduce the incidence of lupus nephritis and skin manifestations, improve lipid profiles, and lower thromboembolic risk. Chronic HCQ treatment can also enhance survivability by minimizing organ damage and osteoporosis from GC use [18]. While the mechanism of action of HCQ is still not fully understood, the therapeutic effects of HCQ are thought to be achieved in part by inhibiting lysosomal activity, reducing inflammatory cytokine production and signaling, and increasing photoprotection against ultraviolet light [16,19]. One of the main side effects of long-term HCQ treatment is retinopathy. Therefore, annual ophthalmologic screening is recommended in patients taking this drug [20]. Other side effects include prolonged QT interval and various dermatologic conditions such as rashes, pruritus, hyperpigmentation, bone marrow toxicity, hypoglycemia, confusion, disorientation, and muscle weakness [19].

GCs are also one of the hallmarks of SLE treatment, especially in mitigating flares of disease activity. They are often used to induce remission and may be used in conjunction with other medications as maintenance therapy. GCs can reduce inflammation and immune activation and dampen SLE activity fairly quickly [16,17]. This is achieved largely by the GCs directly binding to an intracellular receptor, which can alter the transcription of various genes. This effect reduces the production of inflammation-associated molecules such as cytokines, chemokines, and derivatives from arachidonic acid [21]. While GC therapy can be very useful in SLE treatment, its side effects place limitations on the duration and dose of safe treatment. Major adverse effects of chronic GC treatment include osteoporosis, dysfunction of the hypothalamic-pituitary axis, hyperglycemia, weight gain, fat redistribution, muscular weakness, poor wound healing, and skin striae, among others [22]. Due to the large amount of significant adverse effects, it is essential to attempt to restrict use or completely discontinue GC therapy depending on the disease activity or duration of treatment. While GCs play a major role in SLE treatment and can be lifesaving, they are also associated with significant adverse effects, which can lead to increased morbidity and mortality [17].

NCI can also be an essential therapy in the treatment of SLE. These drugs affect B cells and include cyclophosphamide (CYC), mycophenolate (MMF), methotrexate (MTX), and azathioprine (AZA). NCIs are often used in conjunction with HCQ and GCs to reduce SLE activity and are often used as maintenance therapy [17].

CYC targets naïve and pre-switching memory B cells by alkylating DNA and blocking the replication of cells such as B cells. Intravenously administered CYC is often used as a first-line drug and GC to induce remission in patients with proliferative lupus nephritis (PLN) [16]. Oral administration of CYC can result in increased exposure to the drug but carries a higher risk of adverse effects compared to intravenous administration [18]. Common side effects of CYC include alopecia, amenorrhea, hemorrhagic cystitis, nausea, and vomiting. CYC can also cause sterility in both sexes and should not be given to pregnant or breastfeeding patients [23].

MMF is metabolized in the liver into mycophenolic acid, which inhibits the enzyme IMP dehydrogenase, which is essential for T- and B-cell DNA replication. It is administered orally and, like CYC, is often used for treatment in patients with PLN. However, some studies suggest that MMF can lead to more frequent and complete renal remission with fewer side effects. MMF is also thought to be superior to AZA and is often favored for maintenance therapy [16]. MMF should not be given to patients who are pregnant or breastfeeding, and GI issues such as diarrhea, gas, and abdominal pain are common side effects of this medication [24].

MTX is often used in patients with moderate-to-severe SLE for non-renal symptoms such as rashes and arthritis if HCQ and topical steroids are ineffective [16,18]. MTX leads to the inhibition of T-cell activation and downregulation of B cells by inhibiting the enzyme dihydrofolate reductase, resulting in an impairment in DNA replication [25]. Adverse effects of MTX include nausea, vomiting, mucosal ulcers, and, in more severe cases, hepatotoxicity and pulmonary fibrosis leading to a restrictive lung disease. Similar to CYC and MMF, MTX should also not be given to pregnant or breastfeeding patients [25].

AZA is often used in patients with moderate-to-severe lupus as a maintenance therapy. It is a prodrug that interferes with DNA replication in lymphocytes, with dose-dependent effects. However, it has been shown that AZA may increase relapse when used as a maintenance therapy compared to MMF in patients with PLN [26]. Like many of the NCIs discussed, side effects of AZA can include nausea, rashes, and even hepatotoxicity.

FDA-approved biologic treatments for SLE

In addition to the treatments described above, biologic agents are now approved for treating SLE. One of these biologics is belimumab, which is a fully humanized IgG1γ monoclonal antibody that targets soluble forms of B lymphocyte stimulator (BLyS), a co-stimulator needed for the survival and function of B cells [16]. Three types of receptors expressed on B cells interact with BlyS, which include BR3, transmembrane activator and calcium modulator cyclophilin ligand interactor, and B-cell maturation antigen. The affinity between BLyS-BR3, in particular, is strong and promotes the survival of autoantibody-producing B cells by preventing negative selection and apoptosis [27]. This concept was shown in a preclinical experiment involving transgenic mice, which showed that BLyS increased the survival of activated autoreactive B cells. The decrease in self-tolerance resulted in lupus-like autoimmune symptoms [28]. It was also found that patients with SLE had elevated levels of BLyS in their circulation in comparison to individuals without SLE. Therefore, the inhibition of BLyS is critical for improving manifestations of SLE, as it promotes apoptosis of autoreactive B cells [29].

A phase III, double-blinded, clinical trial involving individuals over the age of 18 with seropositive SLE and SELENA-SLEDAI (Safety of Estrogens in Lupus Erythematosus National Assessment-Systemic Lupus Erythematosus Disease Activity Index) scores of at least 6 was conducted to better study the effects of belimumab in patients with SLE. Participants were enrolled and randomly assigned in a 1:1:1 ratio to receive either an intravenous infusion of belimumab, at 1 mg/kg or 10 mg/kg, or a placebo in addition to receiving standard therapy. The infusions took place over one hour on days 0, 14, and 28, and then every 28 days over a period of 48 weeks [30]. By the end of the 52-week trial, patients on belimumab showed a significant reduction in their SLEDAI scores, with 53% of those on 1 mg/kg and 58% on 10 mg/kg achieving a decrease of four points or more compared to only 46% in the placebo group. In addition, patients treated with belimumab reported better quality of life and experienced fewer lupus flares [30]. No significant safety concerns regarding belimumab use were reported in this trial. However, like other biologics, the cost of belimumab is highly high leading to issues in patients being able to afford the medication.

Current EULAR (European Alliance of Associations for Rheumatology) recommendations indicate belimumab use in individuals not responding to HCQ or in patients unable to taper GCs below doses acceptable for chronic use. It is also suggested for use at the onset of active PLN [31]. However, some drawbacks include increased mortality, serious and fatal infections, progressive multifocal leukoencephalopathy, hypersensitivity, and infusion reactions, as well as depression and suicide [31]. Therefore, while belimumab offers a promising option for managing SLE, careful follow-ups with physicians should be a high priority to monitor for possible side effects.

An additional biologic available for the treatment of SLE is anifrolumab. This drug is the second biological agent to be approved by the FDA for the management of SLE. Anifrolumab is a fully human monoclonal antibody that binds to subunit 1 of the type I interferon (IFN) receptor leading to blockage of the effects caused by all type I IFNs, including IFNα, IFNβ, IFNɛ, IFNκ, and IFNω [32]. As noted in lupus, there are elevated levels of IFNα due to stimulation of innate immune receptors via endogenous and exogenous stimuli [33]. The resulting increase in type I IFNs contributes to the survival and activation of autoreactive B cells, exacerbating the autoimmune response. Therefore, blocking the IFNAR may lead to better control of SLE manifestations.

The Treatment of Uncontrolled Lupus via the Interferon Pathway (TULIP) was composed of two phase III clinical trials (LUPUS 1 and LUPUS 2), which assessed the efficacy and safety of anifrolumab in contrast to placebo [34]. These clinical studies were double-blinded, randomized controlled trials in which anifrolumab was administered intravenously every 4 weeks over 48 weeks in patients with moderate-to-severe SLE on standard therapy. To assess the efficacy of anifrolumab, these trials compared the effects of 300 mg of the drug to placebo using the British Isles Lupus Assessment Group-based Composite Lupus Assessment (BICLA), Cutaneous Lupus Erythematous Disease Area and Severity Activity (CLASI-A), and change in GC dosage. Of 726 evaluated patients, a larger number of patients on anifrolumab vs placebo showed improvements in BICLA score starting at week 8 of the trial (p<0.001) [35]. Similarly, greater GC reductions in dosage from baseline were noted in anifrolumab vs placebo at week 20 (p=0.010) [35]. In summary, TULIP phase III trials demonstrate improvements in SLE manifestations compared to placebo.

Like belimumab, anifrolumab may cause fatal infections, hypersensitivity reactions, and malignancies. However, the most commonly reported adverse reactions include nasopharyngitis, upper respiratory tract infections, bronchitis, infusion-related reactions, herpes zoster, and cough [36]. Anifrolumab is indicated in the setting of moderate-to-severe lupus but is not shown in patients with lupus nephritis or severe active nervous system lupus [37]. Given the complex pathology of SLE, finding a precise treatment may require a combination of methods.

Potential new treatments in development for SLE

In addition to the treatments described above, multiple clinical trials are currently underway, exploring therapies targeting SLE. One of these treatments being studied is litifilimab, a subcutaneous IgG1 humanized antibody against blood dendritic cell antigen 2 (BDCA-2) [38]. BDCA2 is a plasmacytoid dendritic cell (pDC) receptor that, when ligated by litifilmab, inhibits the production of IFN-I by human pDCs [38,39]. IFN-I activity has been shown to be chronically elevated in about 50% of patients with SLE, and genetic studies have found that genes related to IFN-I are typically overexpressed in patients with SLE [40]. A phase II clinical trial regarding litifilimab was conducted to assess its safety and efficacy [38]. The trial assigned SLE patients with cutaneous manifestations and arthritis to receive either litifilimab or placebo [38]. The results showed that litifilimab was superior to placebo in reducing arthritis, as fewer joints were inflamed at week 24 compared to baseline in the litifilimab group. However, due to study limitations, no results could be drawn regarding the efficacy of litifilimab in reducing symptoms of cutaneous lupus erythematosus [38]. The adverse events and side effects related to litifilimab are listed in Table 1. Phase III trials involving litifilimab are currently enrolling patient for their study, which will further describe the efficacy and safety of this treatment [38].

Another novel therapy currently in phase III of trials is cenerimod, a sphingosine-1-phosphate receptor 1 (S1P1) receptor modulator [41]. The S1P1 receptor is a crucial receptor subtype in lymphocytes that is believed to play a critical role in lymphocyte migration out of lymphoid organs and into the systemic circulation [41,42]. Because of this, cenerimod functions by preventing lymphocytes from migrating out of lymphoid organs, thus reducing inflammation. When studied in a mice experimental autoimmune encephalitis model, cenerimod decreased proteinuria due to decreasing inflammatory circulating lymphocytes and ultimately increased overall survival [41]. When researchers examined the effects on the brain and kidney specifically, they found that cenerimod led to a decreased incidence of brain pathology in mice compared to that of mice without cenerimod treatment [41]. In clinical trials conducted in human participants with SLE, those who received cenerimod had a statistically significant decrease in biomarkers associated with B-lymphocyte activity, IFN activity, and inflammation [42]. Additionally, the clinical trials found fewer adverse events in cenerimod-treated patients, specifically noting that it did not induce bronchoconstriction or vasoconstriction, unlike other S1P modulators, which have been shown to produce these effects [43]. Cenerimod is currently in phase III of trials, which will further allow researchers to investigate the drug's efficacy in SLE treatment regimens. 

A third treatment in development for SLE is chimeric antigen receptor (CAR) T cells. CAR T-cell therapies have previously been used to treat various hematologic cancers; however, the idea of utilizing them in treating SLE has only recently been explored. CAR T-cell therapies work by collecting T cells from a patient and then “re-engineering” them in a laboratory to produce CARs that bind to specific targets leading to the destruction of said targets when infused back into the same patient [44]. Cluster of differentiation 19 (CD19) is an essential target in the pathophysiology of SLE, as CD19 is a signaling molecule that plays an important role in B-cell development, maturation, and differentiation, making it a good target for CAR-T cells [45,46]. For the novel therapy being studied about SLE, researchers first dampened the immune response of the patients in the study using fludarabine and CYC, resulting in a depletion in the number of circulating lymphocytes [47]. After achieving this, autologous T cells, which were previously harvested from the patients, were transduced with a lentiviral anti-CD19 CAR vector, expanded in vivo, and then reinfused at a rate of 1x10^6^ cells per kilogram body weight. Ultimately, in the five patients (ages 18-24), each participant exhibited loss of the anti-dsDNA marker and other lupus autoantibodies [47]. Additionally, patients received complete remission of disease activity and did not require any immunosuppressive treatment for SLE flares during their anti-CD19 treatment [47]. However, clinical trials for anti-CD19 CAR T-cell therapy are in the early phase I/II and phase I stages, so more data are still necessary to make any definitive comments, but the results in small populations have appeared promising.

Another therapy in development for the treatment of SLE is an anti-toll-like receptor (TLR) 7 monoclonal antibody called DS-7011a. TLR7 is thought to contribute to the development of SLE, so a clinical trial was performed to study the safety, pharmacokinetics, immunogenicity, and pharmacodynamics of DS-7011a when administered via intravenous and subcutaneous doses [48]. Safety was evaluated by recording adverse reactions, pharmacokinetics by measuring plasma DS-7011a, immunogenicity by measuring plasma anti-drug antibodies, and pharmacodynamics by measuring the suppression of IL-6 production in blood. A total of 80 human subjects were divided into three groups: group one received escalating IV doses of DS-7011a, starting with .1 mg/kg and ending with 20 mg/kg; group two received escalating subcutaneous doses beginning with 100 mg and ending with 600 mg; and group three received a single IV dose of 3 mg/kg. It was found that DS-7011a exhibited a long half-life, weak immunogenicity, and strong pharmacodynamic activity in all groups. Adverse events took place in 33% of the subjects who received DS-7011a and in 45% of those who received the placebo. However, the majority of these events were considered minor and not drug-related. Due to there being no serious adverse reactions, deaths, or discontinuations due to adverse reactions, the trial found that DS-7011a was safe and well tolerated across all groups. This study of DS-7011a was the first human trial of the drug and exhibited promising properties for the treatment of SLE. Currently, DS-7011a is undergoing further trials in patients with SLE with a dosing regimen of 20 mg/kg every 4 weeks for 12 weeks [48]. However, a potential downside of this study is that the subjects were mainly of Japanese descent. While this is beneficial for the development of DS-7011a in Japan, further trials, including those involving other groups, will be needed to corroborate the findings of this study and further study the efficacy of DS-7011a in the treatment of SLE (Table 1).

**Table 1:** Potential new treatments under development for SLE BDCA2, blood dendritic cell antigen 2; CAR, chimeric antigen receptor; CD19, cluster of differentiation 19; CRS, cytokine-release syndrome; DNA, deoxyribonucleic acid; ICANS, immune effector cell-associated neurotoxicity syndrome; SLE, systemic lupus erythematosus; TLR7, toll-like receptor 7

Name of treatment under study	Phase of trial	Mechanism of action of treatment	Adverse effects of treatment	Measurement of efficacy of treatment as seen in clinical studies
Litifilimab [38]	Phase II complete. Currently enrolling for phase III.	Monoclonal antibody against BDCA2 plasmacytoid dendritic cell specific antigen	Diarrhea, nasopharyngitis, urinary tract infection, falls, headache, influenza, herpes zoster, herpes keratitis, viral gastroenteritis	Litifilimab resulted in a significantly greater reduction in joint inflammation in patients with SLE compared to placebo, though it did not significantly reduce cutaneous manifestations.
Cenerimod [41,43]	Phase III	Sphingosine-1-phosphate receptor 1 modulator	Abdominal pain, headache, lymphopenia, nasopharyngitis, cholecystitis, chronic pancreatitis, post-cholecystectomy syndrome	Cenerimod resulted in significantly greater reduction of biomarkers such as anti-double stranded DNA antibodies in patients with SLE compared to placebo.
Anti-CD19 CAR T-cell therapy [47]	Some phase I and phase I/II clinical trials	CAR-T cells engineered to express T-cell receptor with strong affinity for CD-19 antigen	CRS and ICANS. These effects are frequently noted in other CD19 CAR-T-cell therapies but were found to be more mild when tried in patients with SLE.	Larger placebo-controlled trials are needed to determine efficacy, however, in the original small case series format, the CAR-T cells seemed to help patients with active SLE achieve disease remission.
DS-7011a [48]	Phase Ib/II	Anti-TLR7 monoclonal antibody preventing TLR7 signaling	No serious adverse reactions were noted.	DS-7011a was found to be safe, exhibited weak immunogenicity, strong pharmacodynamic activity, and a long half life, which are encouraging for its use in SLE treatment. However, further studies are needed to confirm these findings.

Two notable mentions for potential new SLE treatments are Janus kinase inhibitors such as baricitinib and rituximab (RTX). Baricitinib is currently used for the treatment of rheumatoid arthritis and in development of the treatment of SLE. Baricitinib has been shown to suppress circulating anti-dsDNA IgG levels and multiple pro-inflammatory cytokines. Multiple trials have shown promising results, and two phase II trials are in progress to assess the efficacy of baricitinib in lupus nephritis [49]. RTX, approved for treatment of B-cell cancers, is a biologic that eliminates B cells from the blood and is often used off label for the treatment of SLE. Clinical trials are currently underway; however, past studies have yielded mixed results. Despite this, many physicians and patients believed RTX is an effective treatment option for SLE [50,51].

## Conclusions

SLE is a serious disease that affects many individuals across the world. This disease is an autoimmune condition with an etiology that is still not fully understood but is thought to be caused by many different factors. Classically, in addition to lifestyle modifications, treatment for SLE consists of HCQ, GCs, and non-GC immunosuppressants. Since these treatments do not often fully alleviate symptoms in patients with SLE and the significant adverse effects these treatments cause, newer treatments are now used in conjunction with those previously mentioned. With the emergence of these new therapies, we hope that patients with SLE may be put on a treatment regimen that effectively controls symptoms while leading to minimal adverse effects.
